# On the Use of the Belladonna in Extracting the Cataract

**Published:** 1801-10

**Authors:** 

**Affiliations:** Surgeon to the Leicester Infirmary


### *On the Use of the Belladonna in extracting the Cataract, by* Mr. Paget, Surgeon to the Leiceſter Infirmary.

To the Editors of the Medical and Physical Journal.

Gentlemen,

In one of the numbers of your Journal, (vide Medical and Phyſical Journal, vol. iii. p. 367,) we are informed that Dr. Reimarus of Hamburgh, had obſerved a remarkable dilatation of the pupil of the eye to take place conſequent to the application of diluted extract of Belladonna, and that it had been adopted with ſucceſs by Dr. Graſſmeyer of the ſame place, as an expedient to prevent injury being done to the iris during the operation of extracting the cataract. In a ſubſequent number there is a paper by Mr. Wainwright, a reſpectable ſurgeon of Dudley, with an account of ſome obſervations he had made on the uſe of the Belladonna as applied to the eye, which confirmed the favorable report of Reimarus, and induced me to give it a trial as ſoon as an opportunity ſhould occur. I have accordingly uſed it lately in three caſes, previous to the extraction of the cataract; and although the reſult has not quite equalled my expectations, yet I am convinced it will be found of conſiderable uſe when experience ſhall have decided to what extent the application may be carried, without danger of evacuating the vitreous humor from too great a dilatation of the pupil. In the firſt caſe which occurred, I wiſhed to make an experiment of the effects ſome days previous to the operation; and as no intimation was given in either of the accounts above alluded to, of the ſtrength of the preparation employed, that important point was yet to be learned. Accordingly, I diffuſed 4 grains of the extract procured from Apothecaries Hall in ℥ j. of of water, and six drops of this mixture were dropped into one of the eyes. The application gave no pain, nor produced any apparent irritation; but in about half an hour the iris was perfectly invisible, and the whole circumference of the opake crystalline completely in view. The effect continued gradually diminishing after this time, but the pupil was not reduced to the size of that of the other eye in less than three days. When the time was fixed for the operation, I ordered three drops, of the same strength, to be put into the eye half an hour beforehand. Immediately on the application of the knife to the cornea the iris shewed that its irritabilty was not destroyed, for a contraction of the pupil immediately took place, but not to its natural size, so that plenty of room was left for passing the knife, and compleating the incision without injury to the iris.

The other cases were nearly similar in their result, the orbicular fibres of the iris were in a degree paralyzed by the diluted extract, but I did not find that it had any effect in quieting the muscles of the eye, or that it at all tended to prevent the rolling of the eye during the operation. I think this application seems particularly well adapted to the examination of the cataract, previous to the operation, especially when we wish to ascertain whether the capsule is opake, or only the lens itself; and probably we may find it of use in many other states of the eye, where an accurate examination of the parts posterior to the iris is necessary. I am, &c.

*Leicester, Sept.* 20, 1801. THO. PAGET.

---

